# Abnormalities of the Amygdala in schizophrenia: a real world study

**DOI:** 10.1186/s12888-023-05031-0

**Published:** 2023-08-22

**Authors:** Guangen Zheng, Yang Zhou, Jieming Zhou, Shuting Liang, Xiaoling Li, Caixia Xu, Guojun Xie, Jiaquan Liang

**Affiliations:** 1 Department of Psychiatry, The Third People’s Hospital of Foshan, Guangdong, People’s Republic of China; 2 Nanhai Public Health Hospital of Foshan City, Guangdong, People’s Republic of China

**Keywords:** Schizophrenia, Amygdala, MRI, Cognition, PANSS, RBANS

## Abstract

**Background:**

Amygdala plays an important role in schizophrenia (SC), but its mechanisms are still unclear. Therefore, we investigated the relationship between the resting-state magnetic resonance imaging (rsMRI) signals of the amygdala and cognitive functions, providing references for future research in this area.

**Methods:**

We collected 40 drug-naïve SC patients and 33 healthy controls (HC) from the Third People’s Hospital of Foshan. We used rsMRI and the automatic segmentation tool to extract the structural volume and local neural activity values of the amygdala and conducted Pearson correlation analysis with the Positive and Negative Syndrome Scale (PANSS) and the Repeatable Battery for the Assessment of Neuropsychological Status (RBANS) scores. Finally, we compared the clinical data, as well as the volume and functional changes of the amygdala in SC patients before and after treatment.

**Results:**

Compared with HC, SC had widespread cognitive impairments, significant abnormalities in left amygdala function, while the reduction in volume of SC was not significant. Further Pearson correlation analysis with Bonferroni correction showed that only Immediate memory (learning) was significantly negatively correlated with fractional amplitude of low-frequency fluctuation (FALFF, r = -0.343, p = 0.001, p’ = 0.014 (Bonferroni correction)). When compared and analyzed the data difference of SC before and after treatment, we found that immediate memory and delayed memory of SC showed varying degrees of recovery after treatment (t_learning_ = -2.641, p_learning_ = 0.011; t_story memory_ = -3.349, p_story memory_ = 0.001; t_list recall_ = -2.071, p_list recall_ = 0.043; t_story recall_ = -2.424, p_story recall_ = 0.018). But the brain structure and function did not recover.

**Conclusion:**

There was significant dysfunction in the amygdala in SC, and after conventional treatment, the function of the amygdala did not improve with the improvement of clinical symptoms and cognitive function.

## Introduction

Schizophrenia (SC) is a common mental disorder characterized by emotional, cognitive, and behavioral disturbances that lead to confusion between reality and fiction, and ultimately affect an individual’s social and quality of life [1].

Amygdala plays an important role in SC [2–4], affecting the onset and clinical symptoms of the disorder in the following ways.

Emotional processing: The amygdala was the central hub for processing emotions, especially negative emotions, and was involved in regulating emotions such as fear and anxiety [5, 6]. In SC, the amygdala may exhibit abnormalities in emotional regulation, manifesting as emotional discomfort, emotional blunting, and others [3, 7].

Memory and association: It was connected to brain regions such as the hippocampus and was involved in encoding and storing memories [8]. SC patients might exhibit symptoms such as memory impairment and loosening of association, which might be related to abnormalities in the amygdala [2].

Executive control: Amygdala was closely linked to the prefrontal cortex and inferior frontal cortex [9] and was involved in human executive control functions. Patients with SC may experience impaired executive control functions, which had been linked to structural abnormalities in the amygdala [2]. These abnormalities manifested as difficulties in suppressing impulses and self-regulation [10–12].

In addition, some studies had also shown that there were structural and functional abnormalities in the amygdala in SC, such as a decrease in the volume of the amygdala and a reduction in neuronal activity [2, 13, 14]. These abnormalities might be related to the occurrence and manifestation of SC. However, the current studies of the structural and functional changes of the amygdala in SC was inconsistent. The establishment of a causal relationship between changes in cognitive function and alterations in both the structure and function of the amygdala remains uncertain. Therefore, investigating the role of structural and functional changes in the amygdala before and after treatment in individuals with SC can provide valuable insights into the underlying pathological mechanisms of SC.

## Method

The participants with SC (n = 40) were recruited from the Third People’s Hospital of Foshan (Foshan Mental Health Center). Inclusion criteria were as follows: (1) met the diagnostic criteria for SC in the Diagnostic and Statistical Manual of Mental Disorders 5 (DSM-5); (2) aged between 18 and 60 years old; (3) education level of at least 9 years (to avoid patients who were unable to understand the assessment); (4) Han ethnicity, right-handedness; and (5) had not used any psychiatric medication prior to data collection; (6) all the subjects had no contraindications for MRI scans, organic brain diseases, physical illnesses, drug (substance abuse) history, traumatic brain injury, or neurological diseases.

The healthy controls (HC) group (n = 33) was recruited from the local community. They had no history or family history of psychosis, which was confirmed through an interview with a psychiatrist. The age, gender, and education level were matched with those of the SC group. And they were of Han ethnicity and right-handedness.

Scale assessments: The severity of the disease was evaluated using the Positive and Negative Syndrome Scale (PANSS) [15]. Cognition of participants was evaluated using the Repeatable Battery for the Assessment of Neuropsychological Status (RBANS) [16], which aimed to assess immediate memory, visuospatial construction, language, attention, and delayed memory function. Higher scores indicate better cognitive function.

MRI scanning (3.0 Tesla, General Electric, United States), data processing and statistics: 3D structure MRI scanning parameters: Time repetition (TR) = 8.6 ms, Echo time (TE) = 3.3 ms, Flip angle (FA) = 12°, Field of view (FOV) = 256 mm*256mm, matrix = 256*256, layer thickness = 1 mm, layer spacing = 0 mm, slice number = 172. MRI scanning parameters of resting brain function: TR = 2000 ms, TE = 30 ms, FA = 90º, FOV = 240 mm*240 mm, matrix = 64*64, layer thickness = 4 mm, number of layers = 36, layer spacing = 1 mm, Continuous collection of 250 time point data. Like our previous research [13, 17], SPM8 (http://www.fil.ion.ucl.ac.uk/spm), cat12 (http://www.neuro.uni-jena.de/cat12), and the Data Processing Assistant for Resting-State fMRI DPARSF (http://rfmri.org/dpabi) software were used to preprocess MRI data. Brain structure MRI data were mainly used to measure the volume of amygdala gray matter (calculated according to the Automated Anatomical Labeling (AAL) atlas [18]). The analysis and processing of brain functional MRI data included measuring the local neural activity of amygdala with the fractional amplitude of low-frequency fluctuations (FALFF).

The first MRI data collection and scale assessments for all participants were completed within a day and the second evaluation took place at 24–25 weeks. During this time, doctors prescribed relevant antipsychotic medication (primarily second-generation antipsychotics such as Olanzapine (n = 11), Risperidone (n = 10), Paliperidone (n = 8), Lurasidone (n = 5)) based on the patient’s condition, without conducting psychotherapy. After the testing, participants would receive a transportation subsidy of 300 yuan.

### Statistical analyses

Statistical Product and Service Solutions 23 (SPSS 23, https://www.ibm.com/analytics/spss-statistics-software, IBM, Amonk, New York, United States) was used to analyze the clinical scale scores. The Kolmogorov-Smirnov test (K-S test) showed that the measurement data of SC and HC groups followed a normal distribution. Independent sample t-tests and chi-square tests were employed to compare clinical data at baseline between groups. Paired sample t-tests were utilized to compare the data of SC before and after treatment. The Statistical Parametric Mapping 8 (SPM8) software was employed to conduct a two-sample t-test, comparing the structure and function of the amygdala between the SC and HC groups. Pearson correlation analysis was also performed to investigate the relationship between amygdala volume/function and clinical data. The resulting p-values were subjected to Bonferroni correction.

## Result

We excluded six patients with severe behavioral disorders (destruction or violence) who were unable to complete the MRI scan. A total of 34 SC and 33 HC ultimately completed all evaluations.

The results indicated that compared with HC, SC had widespread cognitive impairments, significant abnormalities in left amygdala function, while the reduction in volume of SC was not significant. Compared with HC, the PANSS score of SC was higher (t = 15.144, p < 0.001), while the RBANS score was lower (t=-4.746, p < 0.001). Additionally, the left Amygdala exhibited a higher FALFF compared to HC (t = 2.561, p = 0.013). (Table 1)

**Table 1:** Comparison of clinical scale and MRI data between HC and SC

		SC (n = 34)	HC (n = 33)	t / χ^2^	p
Age		41.909 ± 9.448	42.264 ± 11.250	-0.140	0.889
Gender (Male/female)		19/14	25/9	-1.891	0.204
Education (Years)		10.42 ± 3.021	11.53 ± 4.392	-1.197	0.236
PANSS		67.33 ± 14.339	30.09 ± 0.379	15.144	< 0.001*
PANSS (Positive)		13.88 ± 6.294	7.03 ± 0.239	6.345	< 0.001*
PANSS (Negative)		21.09 ± 5.719	7.06 ± 0.239	14.297	< 0.001*
PANSS (General)		32.36 ± 7.141	16.00 ± 0.000	13.365	< 0.001*
RBANS		125.757 ± 32.939	169.076 ± 37.056	-4.746	< 0.001*
Immediate memory (Learning)		11.35 ± 4.996	23.96 ± 6.245	-3.401	0.001*
Immediate memory (Story Memory)		6.30 ± 4.760	11.35 ± 4.996	-3.953	< 0.001*
Visuospatial Construction		15.42 ± 3.783	16.77 ± 2.487	-1.564	0.123
Language		12.27 ± 4.598	17.38 ± 4.419	-4.313	< 0.001*
Attention (Digit Span)		10.91 ± 2.590	13.19 ± 2.333	-3.510	0.001*
Attention (Coding)		29.82 ± 11.151	43.69 ± 14.907	-4.091	< 0.001*
Delayed memory (List Recall)		3.15 ± 2.647	4.96 ± 3.013	-2.453	0.017*
Delayed memory (List Recognition)		17.85 ± 2.476	19.23 ± 1.306	-2.575	0.013*
Delayed memory (Story Recall)		3.24 ± 3.042	6.08 ± 3.149	-3.499	0.001*
Delayed memory (Figure Recall)		8.52 ± 5.357	12.46 ± 4.254	-3.069	0.003*
Volume (Amygdala)	L	0.905 ± 0.104	0.945 ± 0.099	-1.659	0.102
	R	1.019 ± 0.111	1.022 ± 0.275	-0.122	0.903
FALFF (Amygdala)	L	-0.073 ± 0.358	-0.272 ± 0.275	2.561	0.013*
	R	-0.211 ± 0.360	-0.312 ± 0.223	1.381	0.172

PANSS: Positive and Negative Syndrome Scale; RBANS: Repeatable Battery for the Assessment of Neuropsychological Status; FALFF: Fractional amplitude of low-frequency fluctuations; L: left; R: right. * Indicated p < 0.05; Independent-sample t-test, two-tailed.

**Fig. 1:**
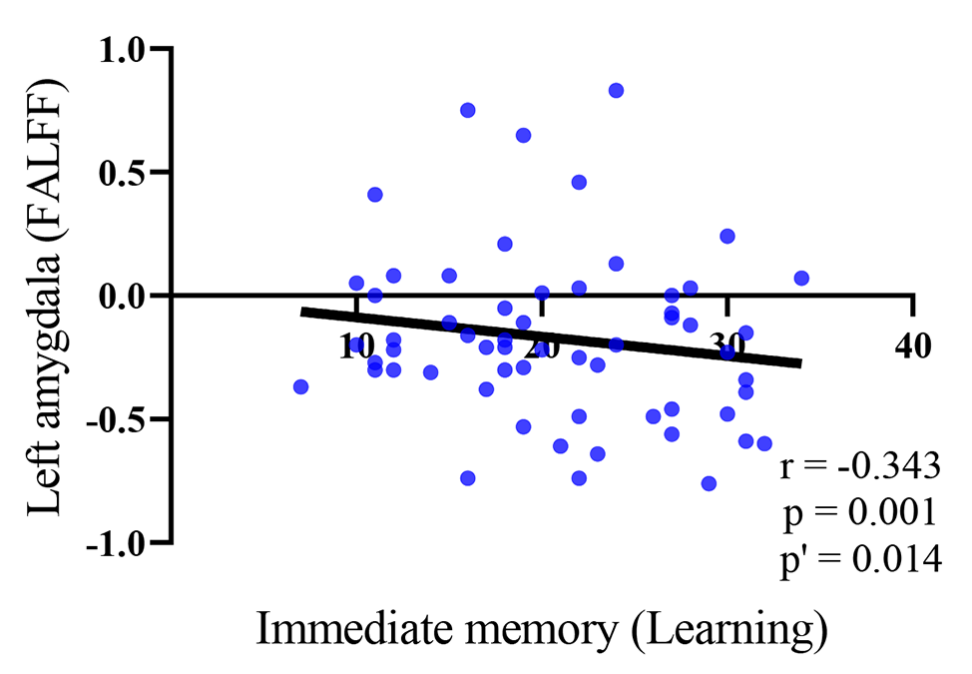
Pearson correlation analysis between the FALFF value of the left amygdala and immediate memory. Immediate memory (learning) was significantly negatively correlated with FALFF (r = -0.343, p = 0.001, p’ = 0.014(Bonferroni correction)); FALFF: Fractional amplitude of low-frequency fluctuations

Pearson correlation analysis was conducted, with Bonferroni correction applied, to further investigate the correlation between disease severity and the left amygdala cognitive function. The results determined that only immediate memory (learning) was significantly negatively correlated with FALFF (r = -0.343, p = 0.001, p’ = 0.014 (Bonferroni correction)), indicating that as FALFF increased, instant learning and memory function decreased. (Fig. 1).

When compared and analyzed the data difference of SC before and after treatment, we found that the overall condition of SC improved after treatment (t_PANSS_ = 3.131, p_PANSS_ = 0.003). However, negative symptoms did not show significant improvement (t_PANSS (negative)_ = 0.994, p_PANSS (negative)_ = 0.324). Immediate memory and delayed memory of SC showed varying degrees of recovery after treatment (t_learning_ = -2.641, p_learning_ = 0.011; t_story memory_ = -3.349, p_story memory_ = 0.001; t_list recall_ = -2.071, p_list recall_ = 0.043; t_story recall_ = -2.424, p_story recall_ = 0.018). Unfortunately, the brain structure and function did not recover. (Table 2)

**Table 2:** Comparison of the disease status, cognition, and resting-state magnetic resonance data of SC before and after treatment

		SC _0−week_	SC _24−week_	t	p
PANSS		67.33 ± 14.339	56.41 ± 12.933	3.131	0.003*
PANSS (positive)		13.88 ± 6.294	10.38 ± 4.467	2.492	0.015*
PANSS (negative)		21.09 ± 5.719	19.59 ± 6.193	0.994	0.324
PANSS (general)		32.36 ± 7.141	26.45 ± 5.047	3.718	< 0.001*
RBANS		125.757 ± 32.939	138.093 ± 39.223	-1.346	0.183
Immediate memory (Learning)		11.35 ± 4.996	22.57 ± 6.155	-2.641	0.011*
Immediate memory (Story Memory)		6.30 ± 4.760	10.46 ± 4.925	-3.349	0.001*
Visuospatial Construction		15.42 ± 3.783	16.46 ± 3.203	-1.147	0.256
Language		12.27 ± 4.598	12.71 ± 3.463	-0.417	0.678
Attention (Digit Span)		10.91 ± 2.590	10.86 ± 2.256	0.083	0.934
Attention (Coding)		29.82 ± 11.151	32.36 ± 10.962	-0.893	0.375
Delayed memory (List Recall)		3.15 ± 2.647	4.54 ± 2.546	-2.071	0.043*
Delayed memory (List Recognition)		17.85 ± 2.476	18.54 ± 3.574	-0.883	0.381
Delayed memory (Story Recall)		3.24 ± 3.042	5.11 ± 2.936	-2.424	0.018*
Delayed memory (Figure Recall)		8.52 ± 5.357	9.43 ± 4.947	-0.687	0.495
Volume (Amygdala)	L	0.905 ± 0.104	0.900 ± 0.102	0.183	0.426
	R	1.019 ± 0.111	0.980 ± 0.093	1.544	0.127
FALFF (Amygdala)	L	-0.073 ± 0.358	-0.086 ± 0.342	0.282	0.779
	R	-0.211 ± 0.360	-0.145 ± 0.336	1.849	0.073

PANSS: Positive and Negative Syndrome Scale; RBANS: Repeatable Battery for the Assessment of Neuropsychological Status; FALFF: Fractional amplitude of low-frequency fluctuations; L: left; R: right. *Indicated p < 0.05; Paired-sample t-test, two-tailed.

## Discussion

This study focused on the structural and functional changes of the amygdala in the drug-naïve SC. Real-world SC patients were collected, and their symptoms, cognitive functions, and brain MRI data before and after treatment were analyzed. The results suggested that SC patients had extensive cognitive impairment and the function of the left Amygdala was significantly increased. Although the symptoms could be partially recovered with regular treatment, the neuroimaging results showed that the functional abnormalities of the amygdala could not be restored.

SC had widespread cognitive impairments, which were consistent with previous research findings [19, 20] Additionally, we discovered a significant increase in local neural activity in the left amygdala. Previous research has suggested a close relationship between the amygdala and the observed mood dysregulation in SC, with abnormal structure and function of the amygdala being associated with psychotic symptoms [13, 21–24]. Some researchers had indicated that SC patients have reduced volume changes in the amygdala [21, 25, 26], while others had concluded that early and comprehensive treatment could repair brain atrophy in SC patients [27]. However, our study did not find any significant volume differences between SC patients and HC, which may be attributed to our selection of treatment-naïve SC individuals. The participants in our study showed that even if the amygdala function was overactive, it might not have progressed to amygdala atrophy, as seen in chronic SC patients [28–30]. Additionally, the follow-up period in our study was 24 weeks, which might not have been long enough for amygdala atrophy to occur. Therefore, this finding can be interpreted in this context.

Subsequently, we used Bonferroni correction to conduct Pearson correlation analysis between the FALFF signal of the left amygdala and various cognitive functions in untreated SC and HC, and the results showed only a negative correlation with immediate memory (learning) and the FALFF. Previous research showed that the amygdala was closely related to emotion[6, 31, 32]. Under normal conditions, the activation level of the amygdala decreased when encountering emotions like anger or fear. However, emotion regulation was abnormal in SC, and the amygdala function was overactivated instead [33]. Subsequently, many studies had also shown that the amygdala was involved in cognitive processes in the human brain [34, 35], and its dysfunction could seriously impair and affect cognitive and memory levels [36].

When comparing the data of SC before and after treatment, we observed varying degrees of recovery in the severity of illness and cognitive function, particularly in aspects such as immediate memory and delayed memory. Similar to previous studies, patients with SC commonly exhibit severe impairments in memory, which are associated with volumetric abnormalities in the amygdala[37, 38]. And Tetsuya et al. employed fMRI and discovered significant amygdala signal activation during memory processes[39]. However, it is unfortunate that the sample size in this experiment was relatively limited. And there is currently a lack of literature reporting on changes in amygdala following treatment. Additionally, negative symptoms generally considered difficult to recover from, and there was no significant improvement observed in this study [40–42]. Although conventional treatment of SC can improve patients’ symptoms, unfortunately, our results did not show any data on structural or functional recovery of the amygdala. Previous studies had reported that after standardized treatment with antipsychotic drugs, the volumes of the frontal lobe, temporal lobe, and hippocampus in SC could partially recover, but there was no evidence of amygdala volume recovery, and not to mentioned passing through the multiple comparisons [43]. Some scholars had also found no significant functional changes in SC patients treated with olanzapine after 8 weeks of follow-up, similar to the results of our study [44].

All in all, there was significant dysfunction in the amygdala in SC, and after conventional treatment, the function of the amygdala did not improve with the improvement of clinical symptoms and cognitive function. It should be noted that the pathological mechanisms of SC were complex and involved structural and functional changes in multiple brain regions [1, 45]. And this study only focused on the amygdala itself, so caution was still needed when drawing conclusions. Overall, our results still provide important and valuable vertical research references for understanding the abnormal functional mechanisms of the amygdala in SC.

## Data Availability

The datasets generated and/or analyzed during the current study are not publicly available due to confidentiality but are available from the corresponding author on reasonable request.
